# Genome Sequences of Two *Shewanella* spp. Isolated from the Gut of the Sea Cucumber *Apostichopus japonicus* (Selenka, 1867)

**DOI:** 10.1128/genomeA.00674-17

**Published:** 2017-07-20

**Authors:** Hyun-Hee Hong, Hyunjun Choi, Seongmin Cheon, Hyun-Gwan Lee, Chungoo Park

**Affiliations:** a School of Biological Sciences and Technology, Chonnam National University, Gwangju, Republic of Korea; b Department of Oceanography, Marine Ecological Disturbing and Harmful Organisms Research Center, Chonnam National University, Gwangju, Republic of Korea

## Abstract

In this study, we sequenced the genomes of two *Shewanella* spp., newly isolated from the gut of the sea cucumber *Apostichopus japonicus* (Selenka, 1867). The whole-genome sequences reported here will expand the repertoire of genomic information for the members of the genus *Shewanella* and will provide important insights into their roles within microbial communities.

## GENOME ANNOUNCEMENT

The genus *Shewanella* is composed of a diverse group of facultative anaerobic bacteria that are widely distributed in aquatic environments (1–3). Several members of this genus, including* Shewanella colwelliana*, have been shown to produce abundant exopolysaccharides with potential commercial value (4, 5). Moreover, there is increasing evidence that some members, including *S. algae*, are human pathogens (6, 7). The *Shewanella* genus is ecologically and physiologically diverse; thus, genomics will play a major role in understanding the physiological complexity of its members (1–3). Here, we present the draft genome sequences of the strains *S. colwelliana* CSB03KR and *S. algae* CSB04KR, isolated from the gut of the sea cucumber *Apostichopus japonicus* from Geomun-do, Yeosu, Republic of Korea (lat 34.1, long 127.18).

The two isolated pure cultures of *Shewanella* spp. were first identified by 16S rRNA gene sequence analysis using EzTaxon-e (8). Genomic DNA of the harvested *S. colwelliana* CSB03KR and *S. algae* CSB04KR strains was extracted using the phenol-chloroform extraction method. Their genomes were sequenced using the Illumina HiSeq 4000 platform, employing paired-end reads (2 × 151 bp) prepared with the Accel-NGS 2S PCR-free library kit (Illumina, San Diego, CA, USA) according to the manufacturer’s instructions. A total of 7,865,394 and 7,492,116 raw sequence reads were generated from *S. colwelliana* and *S. algae*, resulting in 255× and 235× coverages of the genomes, respectively. The raw sequence reads were filtered using Trimmomatic (9) for quality control (trimming adapter and low-quality sequences), and all trimmed sequence reads were *de novo* assembled with three different assemblers, including Abyss version 1.9 (10), SOAPdenovo2 (11), and Velvet version 1.2.10 (12). Finally, qualifying scaffolds from the three assemblies were integrated using CISA version 1.3 (13), which showed an outstanding performance compared to the results from each assembler. From the assessment of the *de novo* assembly using QUAST version 4.1 (14), the assembled draft genome for *S. colwelliana* CSB03KR consisted of 58 scaffolds, covering 4,655,471 bp, the longest of which was 594,967 bp, and had a 45.4% GC content and an *N*_50_ of 241,596 bp. The draft genome for *S. algae* CSB04KR also consisted of 64 scaffolds, covering 4,803,356 bp, the longest of which was 301,346 bp, and had a 53.0% GC content and an *N*_50_ of 149,689 bp.

Genome annotation was performed with the RAST version 2.0 automatic annotation server (15), using the default options. The annotated *S. colwelliana* CSB03KR genome contains 4,336 genes, including 4,244 protein-coding sequences classified in 514 subsystems, 3 5S rRNAs, 4 SSU rRNAs, 3 LSU rRNAs, and 82 tRNAs. The annotated* S. algae* CSB04KR genome contains 4,358 genes, including 4,256 protein-coding sequences classified in 526 subsystems, 8 5S rRNAs, 1 SSU rRNA, 2 LSU rRNAs, and 91 tRNAs. In summary, the complete genomes of *S. colwelliana* CSB03KR and *S. algae* CSB04KR sequenced in this study will provide comprehensive insights into the microbial ecology of the gut in sea cucumbers.

### Accession number(s).

The complete genomes for *S. colwelliana* CSB03KR and *S. algae* CSB04KR have been deposited in DDBJ/ENA/GenBank under the accession numbers MCBT00000000 and MBFW00000000, respectively.
